# A study of brain function changes in patients with trigeminal neuralgia of different laterality based on rs-fMRI

**DOI:** 10.22514/jofph.2025.015

**Published:** 2025-03-12

**Authors:** Li Li, Hai Du, Xin-Yi Li, Chen-Ming Yu, Bing-Bing Huang, Zi-Tang Ma, Rui Li

**Affiliations:** ^1^Department of Radiology, Xuzhou Cancer Hospital, 221005 Xuzhou, Jiangsu, China; ^2^Department of Radiology, Ordos Central Hospital, 017000 Ordos, Inner Mongolia, China; ^3^Department of Radiology, Jiangxi Maternal and Child Health Hospital, 330006 Nanchang, Jiangxi, China; ^4^Department of Clinical Medicine, Jining Medical University, 272011 Jining, Shandong, China; ^5^Department of Radiology, Jining No. 1 People’s Hospital, 272011 Jining, Shandong, China

**Keywords:** Trigeminal neuralgia, Functional magnetic resonance imaging, Amplitude of lowfrequency fluctuations, Regional homogeneity

## Abstract

**Background**: This study employed resting-state functional magnetic resonance imaging (rs-fMRI) to examine alterations in the brain’s spontaneous activity during rest in patients with trigeminal neuralgia (TN) affecting different sides of the face. **Methods**: We included 30 cases each of right-sided TN (R_TN), left-sided TN (L_TN), and healthy controls (HC). We analyzed changes in amplitude of low-frequency fluctuations (ALFF) and regional homogeneity (ReHo) values between L_TN and R_TN groups in comparison to HC. We also explored relationships between disease duration, visual analog scale scores, and ALFF/ReHo values in significant brain regions. **Results**: Relative to HC, L_TN exhibited increased ALFF values in the left superior temporal gyrus and reduced values in the bilateral middle frontal gyrus. Elevated ReHo values were observed in the left cerebellar Crus2 region, while decreased values were identified in the bilateral middle frontal gyrus and left dorsolateral superior frontal gyrus. In R_TN, ALFF values increased in the left precentral gyrus and decreased in the right middle frontal gyrus; ReHo values remained unchanged. Correlation analysis indicated positive associations between disease duration and ALFF value of left superior temporal gyrus, as well as ReHo value of left cerebellar Crus2 region in L_TN. **Conclusions**: This research indicated that both left and right TN patients exhibited changes in spontaneous brain activity during rest. These alterations predominantly occurred contralateral to the pain. These identified brain regions are implicated in pain perception, regulation, and emotional processing, suggesting their relevance to the modulation and adaptive changes of the human brain in response to trigeminal neuralgia.

## 1. Introduction

Trigeminal neuralgia (TN) ranks among the most prevalent neuropathic pain conditions, characterized by sudden, severe, and brief pain episodes within the distribution area of one or more trigeminal nerves [1]. Some patients will develop persistent pain with the duration of the disease [2]. Statistics reveal an incidence of TN ranging from 2.1 to 27 cases per 100,000 individuals [3]. The right side is more frequently affected, with a left-to-right ratio of approximately 1:2 [4]. Females exhibit a higher prevalence than males, and the peak occurrence is typically between the ages of 50 and 60, with incidence increasing as individuals age [5]. Prolonged episodes of acute pain not only disrupts patients’ daily activities but also exerts a significant psychological impact, potentially resulting in symptoms such as anxiety and depression. Therefore, a comprehensive understanding of the underlying pathophysiology is pivotal for improving patient management.

Currently, the pathogenesis of TN remains elusive, with the most widely accepted theory being the vascular compression theory [6]. This theory suggests that nerve fibers at the entry point of the trigeminal nerve root experience compression from blood vessels, leading to demyelination [7]. Consequently, exposed neuron axons display a lowered excitation threshold. Even a minor stimulus can trigger excessive electrical activity in the neuron, subsequently exciting adjacent resting neurons. This synchronized propagation of abnormal discharges leads to central transmission, resulting in the generation of pain signals. This mechanistic insight has popularized microvascular decompression as an effective TN treatment. However, recent clinical studies have disclosed that roughly 13% of patients lack identifiable compressing vessels during surgery [8], and about 5% experience incomplete pain relief or recurrence following microvascular decompression [9], underscoring that vascular compression and demyelination might not universally underlie trigeminal neuralgia.

With the rapid advancement of neuroimaging techniques, resting-state functional magnetic resonance imaging (rs-fMRI) has progressively found application in investigating various mental disorders such as insomnia [10], schizophrenia [11], and Parkinson’s disease [12]. rs-fMRI indirectly captures neuronal spontaneous activity by detecting blood oxygen level-dependent (BOLD) signals. It proves useful in studying early-stage brain function abnormalities, offering simplicity, noninvasiveness, and reproducibility as benefits [13]. Amplitude of Low Frequency Fluctuation (ALFF) and Regional homogeneity (ReHo) are two widely employed metrics in brain function analysis, serving as effective tools for examining distinct aspects of initial signals on a whole-brain scale. ReHo primarily assesses the consistency of BOLD signals between voxels and their neighboring voxels in the time series, reflecting the synchronization of neuronal activity within local brain regions [14]. The higher ReHo values represent better consistency between the corresponding voxel and adjacent voxels, while a decrease suggests a reduction in the spontaneous activity consistency of the local neurons. ReHo, which uses noise reduction in both temporal and spatial domains, demonstrates good stability and serves as a reliable indicator of reflecting the coordination between local neuronal activity and the surrounding voxels. ALFF, on the other hand, quantifies the summation of spectral amplitudes of voxel signals within the low-frequency range (typically 0.01–0.08 Hz). This measure assesses the strength of spontaneous neuronal activity within specific brain regions by capturing the amplitude of low-frequency fluctuations associated with that activity [15]. When abnormal activity of certain neurons causes the BOLD signal to deviate from the baseline, it leads to an increase in the ALFF value, indicating an enhanced spontaneous brain activity. Conversely, a decrease in the ALFF value reflects weakened spontaneous brain activity. Its measurement and calculation are simple and reliable, making it a valuable indicator of spontaneous nerve activity intensity.

Recent neuroimaging studies in patients with TN have detected abnormalities in the structure and function of specific brain regions. Multiple studies focusing on brain structure have indicated that gray matter volume changes primarily occur in the frontal lobe, basal ganglia system, and cerebellum among TN patients. Additionally, reductions in the gray matter volume of these brain regions have been found to correlate with disease severity [16, 17, 18]. Studies examining brain function have also revealed impairments in TN patients, particularly within the frontal, temporal, and parietal regions, as well as the cerebellum [19, 20]. However, it is worth noting that a study has shown that unilateral pain is the central feature of patients with TN, and the right head and face are more susceptible than the left. Some studies on migraine have found that lateralized pain usually occurs on the left side, which may be related to the fact that the right cerebral hemisphere is less efficient in processing skin sensation than the left cerebral hemisphere [21]. Ahmet [22] found that the left hemisphere of left migraine patients was more likely to have deep white matter hypersensitivity, while right migraine patients were more likely to have deep white matter hypersensitivity in the right hemisphere through the study of the correlation between white matter hypersensitivity and pain lateralization in patients with paroxysmal migraine. In previous studies on changes in brain function in patients with TN, only a limited number of studies have explored TN patients’ pain side separately.

In this study, we employed rs-fMRI to analyze changes in ALFF and ReHo values among patients with unilateral TN affecting both the left and right sides. We further examined the correlation between abnormal ALFF and ReHo values, disease duration, and the visual analogue score (VAS). The aim was to provide new imaging evidence to enhance the understanding of TN’s pathogenesis.

## 2. Materials and methods

### 2.1 Subjects

Patients attending the clinic between September 2021 and January 2023 were divided into three groups: left-sided TN (L_TN), right-sided TN (R_TN), and a healthy control (HC) group matched for age and gender. Each group comprised 30 cases. The study was approved by the hospital’s ethics committee and the subjects were informed and signed an informed consent form. Inclusion criteria were as follows: (i) compliance with the International Classification of Headache Disorders criteria (third edition) for the diagnosis of TN; (ii) presence of unilateral pain involving one or more branches of the trigeminal nerve (ophthalmic, maxillary, and mandibular branches) within the distribution area; (iii) after drug treatment is ineffective, or cannot tolerate the medication’s side effects and severely affects the work and life of the patients with acute attack; (iv) no history of prior treatment with radiofrequency ablation or microvascular decompression prior to scanning; and (v) the experience of right-sided sharp hand. Exclusion criteria included: (i) contraindications to magnetic resonance scanning; (ii) compliance with the International Classification of Headache Disorders criteria (third edition) for the diagnosis of secondary trigeminal neuralgia; (iii) other types of head and facial pain; (iv) serious physical or mental illness. Clinical information of patients was collected, and pain intensity was evaluated using a VAS ranging from 0 to 10.

### 2.2 Image acquisition

Individuals underwent examination using a 12 channel head coil on a 3T MR scanner (WAGNETOM Trioa Tim, Siemens Healthcare, Erlangen, Bavaria, Germany) within the Verio system. Participants were positioned supine and provided with earplugs to attenuate noise. A soft foam cushion was placed under the head to minimize movement and patients are told to be awake during scanning and to avoid intentional thinking as much as possible. To rule out intracranial space-occupying lesions, standard T1-weighted imaging, T2-weighted imaging, and T2-fluid-attenuated inversion recovery and diffusion weighted imaging sequences were performed. We used gradient-recalled echo-planar imaging (GRE-EPI) and three-dimensional T1 weighted imaging (3D-T1WI) sequences to acquire functional and high-resolution structural images. The GRE-EPI sequence parameters were as follows: repetition time (TR) = 2000 ms, echo time (TE) = 30 ms, flip angle = 90°, slice thickness = 3 mm, field of view (FOV) = 220 mm × 220 mm, voxel size = 3 mm × 3 mm × 3 mm, slice count = 34, matrix = 64 × 64, and scanning duration 8 min 6 s. The 3D-T1WI sequence parameters were as follows: TR = 1900 ms, TE = 2.52 ms, flip angle = 7°, slice thickness = 1 mm, FOV = 256 mm × 256 mm, voxel size = 1 mm × 1 mm × 1 mm, slice count = 176, matrix = 256 × 256, and scanning duration 4 min 26 s (Table 1).

**Table 1.** GRE-EPI and 3D-T1WI sequences parameters.

Parameters\Sequence	3D-T1WI	GRE-EPI
TR	1900 ms	2000 ms
TE	2.52 ms	30 ms
Matrix	256 × 256	64 × 64
FOV	256 × 256 mm	220 × 220 mm
Voxel size	1 × 1 × 1 mm	3 × 3 × 3 mm
Flip angle	7°	90°
Slice count	176	34
Slice thickness	1 mm	3 mm
Scanning time	4 min 26 s	8 min 6 s

*TR: repetition time; TE: echo time; FOV: field of view; GRE-EPI: gradient-recalled echo-planar imaging; 3D-T1WI: three dimensional T1 weighted imaging.*

### 2.3 fMRI data processing

Initially, raw data were screened using the MRIcron software to exclude images with excessive artifacts. Subsequently, RESTplusV1.2 (http://www.restfmri.net) implemented preprocessing of rs-fMRI raw data on the MATLAB 2013b platform [23]. The steps were as follows: (1) Converting Digital Imaging and Communications in Medicine format to Neuroimaging Informatics Technology Initiative format. (2) Using the middle layer (layer 17) as a reference layer for slice timing. (3) Based on the head movement calibration curve, excluding data with head motion translations more than 3 mm and/or rotations more than 3°. (4) Aligning functional images to standard space. (5) Using 6 mm × 6 mm × 6 mm full-width half-height gaussian smoothing kernel for spatial smoothing of the data. (6) Trend removal. (7) The covariates were analysed using linear regression analysis to eliminate the influence of irrelevant signals such as head movement, cerebrospinal fluid and cerebral white matter on the experiments. (8) Filtering is done through a 0.01 to 0.08 Hz filter.

Preprocessed data after steps (1)~(7) underwent voxel-wise Fourier transform to derive the frequency power spectrum of signal intensity time series. Squaring this spectrum yielded the amplitude of signal oscillations, and averaging the squared spectrum within the 0.01–0.08 Hz range yielded the ALFF value. For statistical analysis, each voxel’s ALFF value was divided by the whole brain’s average ALFF value to compute the normalized ALFF value.

Unsmoothed preprocessed data (1~4, 6~8) underwent calculation of Kendall’s coefficient of concordance (KCC) for each voxel across the entire brain, normalized by the mean ReHo value of the complete brain voxel. Subsequently, the data was smoothed using Gaussian kernels of 6 × 6 × 6 mm^3^ at half-maximum and subjected to statistical analysis.

### 2.4 Statistical analysis

Normality tests were conducted on age, disease duration, and pain intensity measurements. Independent *t*-tests were utilized to compare VAS scores and disease duration between the left and right TN groups. One-Way ANOVA was employed to compare the ages of the three groups. The analysis was carried out using SPSS software (version 26, IBM Corp., Armonk, NY, USA), and results were presented as mean standard deviation. Statistically significant differences were considered at *p *< 0.05.

ALFF and ReHo values of the L_TN and R_TN groups were compared to those of the HC group using SPM12 software (Centre for Human Neuroimaging, University College London, UK). A two-sample *t*-test was conducted with age and gender as covariates, and the results were corrected for multiple comparisons by the family-wise error (FWE) correction method. Differences reaching *p *< 0.01 at the single voxel level and *p *< 0.05 at the clumping level (FWE corrected) were deemed statistically significant. Correlation analysis between ALFF and ReHo values of differing brain regions across groups with disease duration and VAS scores was carried out using Spearman’s correlation analysis in SPSS 26.0 software, with* p *< 0.05 considered statistically significant.

## 3. Results

### 3.1 Demographics and clinical characteristics

No statistically significant differences were observed in age, sex ratio, disease duration, or VAS scores between the three groups. Additionally, there were no significant disparities in disease duration and VAS scores between the left and right patient groups (Table 2).

**Table 2.** Demographic characteristics of all subjects.

Indices	L_TN	R_TN	HC	*p* value
Sample sizes	30	30	30	—
Male/Female	7/23	13/17	10/20	0.259
Age	53.80 ± 10.94	53.70 ± 11.48	51.77 ± 11.68	0.738
Disease duration (yr)	3.04 ± 1.85	2.91 ± 1.67	—	0.777
VAS	5.73 ± 2.37	6.13 ± 2. 30	—	0.976

*L_TN: Left Trigeminal Neuralgia; R_TN: Right Trigeminal Neuralgia; HC: Healthy Control Group; VAS: visual analogue score; “—” indicates that the data has not undergone further statistical processing. The difference with p < 0.05 is statistically significant.*

### 3.2 Differences in ALFF and ReHo values for L_TN patients

In comparison to the HC group, patients with L_TN exhibited elevated ALFF values in the left superior temporal gyrus and reduced ALFF values in the bilateral middle frontal gyrus. ReHo values were increased in the left cerebellar Crus2 region and decreased in the bilateral middle frontal gyrus and left dorsolateral superior frontal gyrus (single voxel level: *p *< 0.01, cluster level: *p *< 0.05, FWE corrected) (Table 3, Fig. 1A).

**Table 3.** Brain regions with altered ALFF and ReHo values in bilateral TN group compared to HC group.

						MNI coordinates		
Clusters		Brain areas	BA	*t*-values	Peak voxels	X	Y	Z
ALFF differences in L_TN and HC:								
	1	Temporal_Pole_Sup_L	13, 38	4.9677	35	−36	21	−15
	2	Frontal_Mid_L	10	−5.0782	37	−36	33	27
	3	Frontal_Mid_R	9, 10	−5.0475	54	33	33	33
ReHo differences in L_TN and HC:								
	4	Cerebelum_Crus2_L	—	4.2673	57	−48	−63	−39
	5	Frontal_Mid_L	9, 10	−6.1533	96	−33	33	18
	6	Frontal_Mid_R	9, 10	−5.6122	97	33	30	30
	7	Frontal_Sup_L	6	−5.7958	71	−18	45	33
ALFF differences in R_TN and HC:								
	1	Precentral_L	6, 9	5.2149	31	−39	3	45
	2	Frontal_Mid_R	9	−5.3391	33	36	15	39

*No brain areas with altered ReHo values were observed in the R_TN group. The presented regions are all statistically significant at the single voxel level p < 0.01, clump level FWE p < 0.05, and their coordinates (X, Y, Z) are provided in standard stereotactic MNI space. Abbreviations used: ALFF: Amplitude of Low Frequency Fluctuation; ReHo: Regional Homogeneity; MNI: Montreal Neurological Institute; BA: Brodmann Area. L: left; R: right; L_TN: left-sided TN; R_TN: right-sided TN; HC: healthy controls; Sup: superior; Mid: middle.*

### 3.3 Differences in ALFF and ReHo values for R_TN patients

Patients with R_TN displayed increased ALFF values in the left precentral gyrus and diminished ALFF values in the right middle frontal gyrus when compared to the HC group. No significant alterations in ReHo values were observed (single voxel level:* p *< 0.01, cluster level: *p *< 0.05, FWE corrected) (Table 3, Fig. 1B).

**Fig. 1.** Changes in brain regions of ALFF and ReHo values in bilateral group compared to HC group. (A) Brain regions with altered ALFF and ReHo values in L_TN group (single voxel level: *p *< 0.01, cluster level:* p *< 0.05, FWE corrected). (B) Brain regions with altered ALFF values in R_TN group. No brain areas with altered ReHo values were observed in the R_TN group. The red color indicates elevated regions, while the blue color indicates diminished regions.

### 3.4 Correlation analysis

In the L_TN group, a positive correlation was found between disease duration and the ALFF value of the left superior temporal gyrus (*r *= 0.410, *p *= 0.024), as well as the ReHo value of the left cerebellar Crus2 region (*r *= 0.404, *p *= 0.027) (Table 4, Fig. 2).

**Table 4.** Correlation of ALFF and ReHo values in changed brain regions with VAS and medical history.

			VAS		Medical History	
Clusters		Brain areas	*r*	*p*	*r*	*p*
ALFF differences in L_TN and HC:						
	1	Temporal_Pole_Sup_L	−0.148	0.435	0.410	0.024*
	2	Frontal_Mid_L	0.334	0.072	−0.030	0.873
	3	Frontal_Mid_R	0.087	0.649	0.032	0.866
ReHo differences in L_TN and HC:						
	4	Cerebelum_Crus2_L	−0.226	0.229	0.404	0.027*
	5	Frontal_Mid_L	0.274	0.142	0.153	0.418
	6	Frontal_Mid_R	0.273	0.144	0.080	0.673
	7	Frontal_Sup_L	0.079	0.677	−0.036	0.849
ALFF differences in R_TN and HC:						
	1	Precentral_L	−0.173	0.360	−0.179	0.344
	2	Frontal_Mid_R	0.207	0.273	0.150	0.427

*means p < 0.05, the difference was statistically significant. VAS: visual analogue score; L_TN: left-sided TN; R_TN: right-sided TN; HC: healthy controls; ALFF: Amplitude of Low Frequency Fluctuation; ReHo: Regional Homogeneity; L: left; R: right; Sup: superior; Mid: middle.

**Fig. 2.** Correlation analysis of meaningful brain regions with disease duration and VAS. (A) Positive Correlation Between Disease Duration and ALFF Values in Left Temporal Pole Superior for L_TN (*r *= 0.410, *p *= 0.024). (B) Positive Correlation Between Disease Duration and ReHo Values in Left Cerebelum Crus2 for L_TN (*r *= 0.404, *p *= 0.027). ALFF: Amplitude of Low Frequency Fluctuation; ReHo: Regional Homogeneity; L: left; Sup: superior.

## 4. Discussion

The central feature of patients with TN is unilateral pain, with clinical studies indicating a greater susceptibility of the right side of the face to TN compared to the left. It has been hypothesized that the lateralization of pain in TN may be due to narrower dimensions of foramen rotundum and foramen ovale on the right side, but this hypothesis has not been experimentally validated [24]. This study was based on rs-fMRI technology, to investigate changes in local neural activity during resting state in patients with TN experiencing unilateral head and facial pain. The results indicated that compared to the HC group, several brain regions showed abnormalities in these two indicators, which suggests that there are abnormal activity areas in both TN patients with different affected sides and healthy populations. Therefore, the laterality of pain should also be considered as a critical factor in studies examining brain function in patients with TN. In addition, this study simultaneously employed both ALFF and ReHo methods to investigate aberrant brain activity in patients with TN. These two methods based on different neurophysiological mechanisms offer complementary information about changes in spontaneous brain activity within the patient’s brain affected regions. Together, they provide more pathological and physiological information related to the disease than either method alone, contributing to a better understanding of changes in brain function in patients. Through the effective combination of these two methods, our study’s findings indicate differential changes in ALFF and ReHo values between the two patient groups. Interestingly, ReHo values did not reveal any brain area changes after correction in the R_TN group, even though clinical prevalence of R_TN was higher. This suggests that ALFF might encompass broader changes in brain function response than ReHo values, potentially enhancing its sensitivity to alterations.

Bilateral cerebral hemispheres exhibit both structural [25, 26] and functional asymmetry [27, 28], most notably illustrated by the left hemisphere’s dominance in language processing [29]. Long [30] proposed that normal individuals display bilateral hemispheric lateralization in the sensory control of the body and brain function during disease states. Blum [31] applied distinct metrics to uncover hemispheric lateralization of cortex-cortex interactions during resting activity. Their findings highlighted the asymmetry of bilateral cerebral hemispheres in pain processing, revealing two distinct forms of functional lateralization. The left hemisphere predominantly favors intrahemispheric interactions, particularly within cortical regions associated with language and fine motor coordination. In contrast, cortical areas within the right hemisphere linked to visuospatial and attentional processing demonstrate integrated interactions with both hemispheres. Tsai YH [19] investigated functional brain connectivity and gray matter volume changes in left and right lateral TN patients, observing that gray matter volume reduction in left TN patients primarily localized to the left hemisphere compared to the HC group. In right TN patients, gray matter volume reduction spanned both cerebral hemispheres, with altered brain regions including the thalamus, hypothalamus, and nucleus accumbens areas. Wang [32] revealed significant differences between the left and right cerebral hemispheres in functional networks, particularly in the default mode network specialized in the left hemisphere, the ventral and dorsal attention networks specialized in the right hemisphere, and the frontoparietal network displaying specialization in both hemispheres with distinct network patterns. These outcomes parallel our present study’s findings, where brain function changes in L_TN patients were concentrated in the left hemisphere, while R_TN patients exhibited alterations primarily in the right frontal lobe. This might relate to abnormalities in the frontal network of the right cerebral hemisphere. Hence, we hypothesize that lateralized TN patients experience more pronounced functional changes in ipsilateral brain regions. Additionally, investigating brain functional changes in unilateral TN patients using both methods revealed that L_TN patients exhibited more extensive brain functional changes than R_TN patients. Despite the lower clinical incidence of L_TN, the complexity of brain functional changes in L_TN patients is presumed to be higher. However, the mechanism behind this change still needs to be further investigated.

This study revealed diminished spontaneous activity of localized neurons in the ipsilateral middle frontal gyrus for patients with TN on both sides. This observation suggests reduced neuronal excitability within this region, leading to overall inhibition of the local brain region’s physiological function. In a meta-analysis of abnormal activation in brain regions during trigeminal neuralgia, Yu [33] also detected reduced activation in the middle frontal gyrus among TN patients, aligning with our findings. The frontal lobe, involved in cognitive function, attention, and pain regulation [34], is subdivided into the medial prefrontal lobe, dorsolateral prefrontal lobe, and orbital cortex. The middle frontal gyrus corresponds anatomically to the dorsolateral prefrontal cortex, predominantly engaged in external stimuli evaluation, and intimately linked to cognitive and emotional regulation. Prior investigations highlighted the frontal lobe’s high frequency of structural and functional brain abnormalities in migraine headaches, indicating reduced gray matter volume in the left middle frontal gyrus and bilateral inferior frontal gyrus among migraine sufferers [35]. Additionally, chronic pain is associated with concentration difficulties, cognitive changes, depression, and impaired pain regulation in patients. Dehgan [36] demonstrated that individuals with chronic lower back pain exhibited diminished gray matter volume in the medial frontal lobe, suggesting these changes relate to decreased activation and compromised processing of pain information. These findings may underlie the altered local functional activity in the middle frontal gyrus of TN patients.

The precentral gyrus, a component of the somatomotor center, processes proprioceptive signals from the contralateral skeletal muscle. In a ReHo study of TN patients, Wang [37] identified elevated ReHo values in the anterior central gyrus (M1) and posterior central gyrus for the TN group compared to controls. Desouza [38] observed augmented gray matter volume in the primary somatosensory cortex and motor cortex of patients with TN, suggesting abnormalities in cerebral sensorimotor processing and pain modulation. They proposed that M1 alterations might represent a compensatory strategy to mitigate pain-related facial movement inhibition in TN patients. Our study further detected heightened spontaneous activity of local neurons in the left precentral gyrus for R_TN patients. This finding implies that intracerebral activity in TN patients may synchronize locally with pain modulation. Accordingly, we propose that the primary motor cortex could suppress trigeminal pain responses and inhibit maxillary and facial muscle movements as a pain reduction strategy.

In this study, patients with L_TN exhibited increased ALFF values in the left superior temporal gyrus, situated within the temporal lobe—a region recognized for its intricate involvement in memory, emotion, and other cognitive functions. It plays a pivotal role in multimodal verbal emotion perception [39]. A voxel-based morphometry (VBM) exploration of TN patients revealed diminished gray matter volume in the temporal lobe across all patient groups, which scholars linked to emotion perception. Chronic pain could lead to anxiety and depression, with the temporal lobe implicated in their assessment, integration, and prediction [40]. Thus, the abnormal activity in the superior temporal gyrus among L_TN patients suggests a possible link to the emergence of negative cognitive symptoms and anxiety in these individuals.

Elevated ReHo values were predominantly observed in the left cerebellar Crus_2 region for L_TN patients. This area, located in the cerebellar cortical hemisphere, receives extensive input from the cerebral cortex, playing a role in random movement coordination and motor program encoding [41]. Research by Kelly and Strick [42] highlighted that prefrontal area 46 projects onto the Crus_1 and Crus_2 areas of the cerebellar cortex, establishing connections between the primary motor cortex and prefrontal regions. The cerebellum’s role extends beyond motor control; it’s also associated with integrating somatic sensation information, including nociceptive signals. Several studies [43, 44] on acute and chronic pain have indicated cerebellar activation, aligning with our study’s outcomes. Bocci [45] reported that cerebellar transcranial direct current stimulation (ctDCS) can influence pain perception and cortical activity, implying the cerebellum’s potential as a target for chronic pain treatment. These findings suggest that resting-state functional changes in the cerebellum likely stem from the effects of TN-related pain.

To further investigate the relationship between changes in brain function with disease duration and VAS in TN patients, our study conducted correlation analyses between ALFF and ReHo values in regions of altered brain function. The results showed that the ALFF value of the left superior temporal gyrus and the left cerebellar Crus2 area in the L_TN group were positively correlated with the course of the disease. Based on these results, we hypothesize that with the prolongation of disease duration, L_TN patients’ left superior temporal gyrus and left cerebellar Crus2 area frequently experience functional changes. These recurring functional abnormalities may lead to an exacerbation of the functional alterations in the superior temporal gyrus and left cerebellar Crus2 brain regions, thereby resulting in functional remodeling. For clinical treatment, these two brain regions may provide new imaging based-evidence for a central therapeutic target for patients with L_TN, providing a new direction for the treatment of L_TN patients.

This study acknowledges certain limitations that warrant consideration. Firstly, although the current research refines the study of TN patients by lateralization compared to the previous comprehensive study involving all TN patients, it remains a cross-sectional investigation. Longitudinal studies are required to gain a comprehensive understanding of brain function changes in TN patients, particularly in relation to disease progression and or post-treatment evolution, necessitating further exploration. Secondly, during participant selection, patients who had undergone invasive treatments like microvascular decompression or radiofrequency ablation were excluded, but those treated with oral medications were included without accounting for factors such as medication dosage. Therefore, more research is necessary to ascertain whether such medications have any impact on the observed outcomes.

## 5. Conclusions

In summary, our findings indicate altered spontaneous brain activities during resting-state conditions for both left and right TN patients. These altered brain areas are implicated in pain perception, regulation, and emotional processing, and maybe linked to the regulation and adaptation of the human brain to trigeminal neuralgia. Furthermore, compared to healthy control group, alterations in brain function were predominantly in the left cerebral hemisphere in patients with L_TN and those in R_TN were predominantly in the right cerebral hemisphere, so we speculated that the deviations seen in the brain functions of lateralized TN patients were primarily ipsilateral. Moreover, the progression of the disease amplified the ALFF value in the left superior temporal gyrus and the ReHo value in the left cerebellar Crus_2 region among L_TN patients. This observation leads us to hypothesize that these two brain regions could be particularly relevant to the functional changes seen in the brain of individuals with left-sided trigeminal neuralgia.
